# Full title: peripheral venous catheter complications in children: predisposing factors in a multicenter prospective cohort study

**DOI:** 10.1186/s12887-017-0965-y

**Published:** 2017-12-19

**Authors:** Rim Ben Abdelaziz, Habiba Hafsi, Hela Hajji, Hela Boudabous, Amel Ben Chehida, Ali Mrabet, Khadija Boussetta, Sihem Barsaoui, Azza Sammoud, Mourad Hamzaoui, Hatem Azzouz, Néji Tebib

**Affiliations:** 1 Department of Pediatrics, La Rabta Hospital, Tunis, Tunisia; 2 Université Tunis El Manar, Faculté de Médecine de Tunis; LR12SPO2 les maladies héréditaires du métabolisme investigation et prise en charge, Tunis, Tunisia; 3 Université Tunis El Manar, Faculté de Médecine de Tunis; Military General Directorate of Health, department of Epidemiology and Public Health, Tunis, Tunisia; 4 Université Tunis El Manar, Faculté de Médecine de Tunis; Department of Pediatrics B, Hôpital d’enfants Béchir Hamza de Tunis, Tunis, Tunisia; 5 Université Tunis El Manar, Faculté de Médecine de Tunis; Department of Pediatrics A, Hôpital d’enfants Béchir Hamza de Tunis, Tunis, Tunisia; 6 Université Tunis El Manar, Faculté de Médecine de Tunis; Department of Pediatrics C, Hôpital d’enfants Béchir Hamza de Tunis, Tunis, Tunisia; 7 Université Tunis El Manar, Faculté de Médecine de Tunis; Department of Pediatric Surgery A, Hôpital d’enfants Béchir Hamza de Tunis, Tunis, Tunisia; 8 Department of Pediatrics, La Rabta Hospital Jabbari, 1007 Tunis, Tunisia

**Keywords:** Peripheral venous catheter, Children, Complication, Extravasation, Lifespan, Prevention

## Abstract

**Background:**

Peripheral venous catheterization (PVC) is frequently used in children. This procedure is not free from potential complications. Our purpose was to identify the types and incidences of PVC complications in children and their predisposing factors in a developing country.

**Methods:**

We conducted a prospective observational multicenter study in five pediatric and pediatric surgery departments over a period of 2 months. Two hundred fifteen PVC procedures were conducted in 98 children. The times of insertion and removal and the reasons for termination were noted, and the lifespan was calculated. Descriptive data were expressed as percentages, means, standard deviations, medians and interquartile ranges. The Chi2 test or the Fisher test, with hazard ratios and 95% confidence intervals (CI_95%_), as well as Student’s t test or the Mann-Whitney U test were used to compare categorical and quantitative variables, respectively, in groups with and without complications. The Spearman test was used to determine correlations between the lifespan and the quantitative variables. The Kruskal Wallis test was used to test for differences in the median lifespan within 3 or more subgroups of a variable. Linear regression and logistic binary regression were used for multivariate analysis. A *p*-value <0.05 was considered significant.

**Results:**

The mean lifespan was 68.82 ± 35.71 h. A local complication occurred in 111 PIVC (51.9%) cases. The risk factors identified were a small catheter gauge (24-gauge) (*p* = 0.023), the use of a volume-controlled burette (*p* = 0.036), a longer duration of intravenous therapy (*p* < 0.001), a medical diagnosis of respiratory or infectious disease (*p* = 0.047), the use of antibiotics (*p* = 0.005), including cefotaxime (*p* = 0.024) and vancomycin (*p* = 0.031), and the use of proton pump inhibitors (*p* = 0.004).The lifespan of the catheters was reduced with the occurrence of a complication (*p* < 0.001), including the use of 24-gauge catheters (*p* = 0.001), the use of an electronic pump or syringe(p = 0.036) and a higher rank of the intravenous device in each patient (*p* = 0.010).

**Conclusions:**

PVC complications were frequent in our pediatric departments and are often associated with misuse of the device. These results could engender awareness among both doctors and nurses regarding the need for rationalization of the use of PVC and better adherence to the recommendations for the use of each drug and each administration method.

## Background

Peripheral venous catheterization (PVC) is one of the most frequently used procedures in hospitalized children. It is estimated that more than 80% of patients undergo this procedure during hospitalization [1]. In addition to the administration of intravenous fluids, drugs, blood products and parenteral nutrition, peripheral venous catheters are also inserted prophylactically before procedures and in unstable patients for emergency use [2].Despite its frequent use, intravenous cannulation is not without risks. It is a leading source of procedure-related pain in the hospital [3, 4].Complications such as clotting, occlusion, leakage, infiltration, extravasation, phlebitis and infection can also occur [5]. In neonatal intensive care units, local complications occur in up to 97%of PVCs [6–9], and the catheters have a median lifespan of 23–40 h [6, 10]. Studies in pediatric departments have reported a lower incidence of complications in approximately a quarter of PVC cases [11–13], with catheters having a median lifespan ranging from 29 to 60 h [13–16].

Various risk factors of local PVC complications have been identified in these studies, which may be related to patient characteristics (a younger age [15, 16]), underlying conditions such as diabetes or cancer [12], the nature of drugs or fluids used (antibiotics, corticosteroids, hypertonic solution, etc) [6, 17], the site of insertion (the antecubital fossa [11, 18],wrist [15] and ankle [16]), the characteristics of the catheter(a smaller gauge [15]), drug and medication administration methods (infusion pumps [12]), and the means of maintaining and securing the PVC (the use of flushes and splints improves PVC patency [15, 19]).

In recent years, significant technological innovations have been developed to promote the insertion use and means of securing peripheral intravenous catheters (PIVC). Cannula insertion can be facilitated with vein visualization devices or ultrasound guidance [20–22]. Medical-grade tissue adhesives (cyanoacrylates) and antimicrobial-impregnated dressings have shown promise in reducing local and infectious complications associated with intravenous therapy [23–25]. According to recent data, clinical practice and international best practice recommendations for the use, care and maintenance of PVCs have considerably changed [26].

In Tunisia, as in many other developing countries, new technologies are not available because of their high cost. Even basic products such as chlorhexidine or transparent film dressings are not routinely used in pediatric units. Therefore, the available data on PVC complications in children, their risk factors and recommendations for best practice are not systematically generalizable to our circumstances.

Our study aimed to (i) provide data on the lifespan of PIVC in children, (ii) describe the incidence and the types of complications of PVC in Tunisian pediatric departments, and (iii) identify any significant risk factors for PVC failure.

## Methods

### Study design and settings

We conducted a prospective, multicenter study over a period of 2 months, including April and May of 2015.Two university hospitals in Tunis participated: The Children’s Hospital Béchir Hamza of Tunis (CHBH), the pediatric Hospital in Tunisia, and La Rabta Hospital (LRH),a predominantly adult hospital hosting a pediatric department specialized in inherited metabolic diseases. In total, four pediatric departments and one pediatric surgery department participated in the study. The overall hospitalization capacity of these departments is 260 beds. Only the pediatric department of LRH is a mother-child ward. In the other wards, children are hospitalized by themselves and mothers are allowed to visit for a few hours during the day.

### Sample and recruitment

All patients admitted to the hospital and requiring at least one PIVC were included. Children referred from another department with a pre-existing PIVC were excluded. Any children who underwent PIVC placement later during hospitalization were included. Children transferred out with a PIVC in place were also noted, but they were not followed up in the wards. These PVCs were labeled as non-complication cases.

We calculated that a sample of at least 200 PVCs was clinically practical and sufficient to provide a heterogenous sample containing all important factors that contribute to complications and would be large enough to overcome potential biases [27].Therefore, we used a convenient study period of 2 months based on feasibility and the number of patients treated in the participating wards.

Study participation was initiated by a member of the research team during routine morning visits who collected baseline data and attached the data collection form to the patient’s medical file.

Each patient was given a case number, and each catheter was numbered in the order of the time of insertion.

### Outcome variables

The primary outcomes were (i) the lifespan of the PIVC (calculated from the time of insertion to the time of removal) and (ii) the occurrence of a complication (occlusion, infiltration, phlebitis, necrosis, displacement or infection). Both parameters were defined by clinicians per their standard clinical judgment. No formal assessment tool was used. The secondary outcomes were variables with potential significant associations with PVC complications, including patient characteristics (age, gender, medical diagnosis), indication for PVC, PIVC gauge, the site of insertion, medications and fluids, and administration method. These specific variables were chosen based on previous literature reporting risk factors of PVC complications in children with consideration for the feasibility of their measurement given the limited human resources dedicated to this study.

### Data collection

Details related to the primary and secondary outcome variables were collected on a paper form designed for this study. Two research nurses dedicated to the study completed the forms during business hours based on information from monitoring sheets used in each ward. Every day, each nurse went into one of the two hospitals on an alternating basis to ensure a daily presence in the study sites.

### Catheter placement and monitoring

The participating hospitals do not have special intravenous insertion teams, so all PIVCs were inserted by nurses who had completed PIVC insertion training. Nurses were not aware of the study. Alcohol 70%was used to decontaminate the skin and devices involved in catheter manipulation. Both 24- and 22-gauge Teflon cannulae were used (B-CAT I.V. cannula, BIÇAKCILAR®, Istanbul, Turkey and PRIMAFLON® I.V cannula, Med Devices Lifesciences Ltd., London, UK).Ultrasound and vein visualization devices were not available and were not used for PIVC insertion during the study. Tape dressings (Neoplast ® universal tape and universal tape perforated with zinc oxide, Adhe-elss.a. Sousse Tunisia) were used for the insertion site and the catheter hub. When necessary, additional nonsterile tape, bandages and splints were used. No extension set was used between the PIVC and devices used for infusions or syringes for the administration of boluses. The infusions and medications were given by either the gravitational method (with or without a volume-controlled burette (VCB)) (Alaris® GW volumetric pump, CareFusion, Hampshire, UK and Romsons ® vented infusion set, Romsons Junior, Agra India and Infusion burette SO.F.A.P TN, Tunis, Tunisia), infusion pumps or electronic syringes (depending on their availability) (Lifepum ® Syringe pump, Well Kang Ltd., Dublin, Ireland, Perfusor ® compact, Braun and Phoenix® syringe pump, Foures, GRADIGNAN - France), or bolus injection. PIVCs were flushed with normal saline solution before use. The insertion site was assessed during nursing rounds or if a sign (from the patient himself or his mother, the medical staff or the infusion device) of potential dysfunction emerged. Dressings were replaced if they were loose or if visible ooze was noticed. In all cases, the catheters were discontinued or replaced based on clinical indications (no routine changes to the catheters).

### Ethical considerations

The study was approved by local Clinical Research Ethics Committee. In our study there was no experimentation or intervention on patients. The manuscript did not contain any recognizable patient data, so a written informed consent was not required. Verbal parental permission was obtained and a subject’s consent was also obtained when appropriate.

### Statistical analysis

The data were processed using SPSS Version 21 software. For categorical variables, we calculated percentages. For quantitative variables, we calculated the means, standard deviations, medians and interquartile ranges (IQRs). For intergroup comparisons, we used the Chi2 test or the Fisher exact test for categorical variables. Hazard ratios (HR) were analyzed with 95% confidence intervals (CI_95%_). For quantitative variables, we used Student’s t-test or the non-parametric Mann-Whitney Utest as indicated. The Spearman test was used to determine correlations between two quantitative variables (such as the lifespan of a PIVC and the age of a patient or the total duration of intravenous (IV) therapy). The Kruskal Wallis test was used to test for differences in the median lifespan within 3 or more subgroups of a variable (such as administration method and study site). For the multivariate analysis, we included variables with a *p*-value < 0.2 in the univariate analysis and any other variable predicted to be a potential risk factor for the outcome variables (the occurrence of complications and PIVC lifespan) based on previous literature. The forced entry method was used to introduce all predictor variables in one block. For the variable occurrence of a complication, we used logistic binary regression and for the variable PIVC lifespan, we used linear regression. A p-value <0.05 was considered significant.

## Results

### Participant characteristics

In this study, 98 children were included. The mean age was 5.2 ± 4.7 years (range 0.1–18). The medical diagnoses groups were respiratory diseases (24.5%), infectious diseases (15.3%), hematological diseases (18.3%), metabolic diseases (20.4%), dehydration (2%), surgery (6.1%), neurological diseases (3.1%) and other diseases (11.2%). The details of the participants’ characteristics are given in Table 1.

**Table 1:** Characteristics of participants (*n* = 98)

	*N* (%)	Median (IQR)
Male gender	49 (50)	
Age (years)		4.5 (6.67)
Site of the study		
Pediatric Department A of CHBH	31 (32)	
Pediatric Department B of CHBH	8 (8)	
Pediatric Department C of CHBH	22 (22)	
Surgery Department A of CHBH	5 (5)	
Pediatric Department of LRH	32 (33)	
Diagnosis		
Infection	15 (15.3)	
Respiratory	24 (24.5)	
Hematology	19 (18.3)	
Metabolic	18 (20.4)	
Dehydration	2 (2)	
Neurologic	3 (3.1)	
Surgery	6 (6.1)	
Other	11 (11.2)	
Duration of hospitalization in the study sites (days)		6 (7.25)
Total duration of intravenous therapy/patient (days)		4.5 (4.75)

*CHBH* Children’s Hospital Béchir Hamza, *LRH* La Rabta Hospital, *IQR* Inter-quartile range

### Catheter characteristics

Two hundred fifteen PIVCs were inserted with a mean of 2.28 ± 1.69 PIVCs/patient (range 1–9). Twenty-two-gauge catheters were used in 53.7% of the cases. The insertion site was in the hand in 82.2% of the cases. The main indications for PVCs were drug administration in 67.8% of the cases, infusion in 24.8% of the cases and blood product transfusion in 7.5% of the cases. The details of the device characteristics are given in Table 2. The gravitational method (with or without VCB) was predominantly used, accounting for 77.7% of the cases. The total duration of IV therapy was significantly longer when a VCB was used (14.91 ± 4.01 days) compared to the gravitational method (7.62 ± 4.01 days) or an infusion pump (6.23 ± 4.54 days) (*p* < 0.001).

**Table 2:** Characteristics of devices (*N* = 215)

	N (%)	Median (IQR)
Number of devices/patient		2 (2)
Indwelling time (hours)		72 (48)
Site of PVC insertion		
Dorsum of the hand	170 (78.8)	
Wrist	2 (1)	
Antecubital fossa	3 (1.5)	
Lower arm	2 (1)	
Foot	37 (17.2)	
Head	1 (0.5)	
Cannula gauge		
22G (blue)	115 (53.5)	
24 G (yellow)	100 (46.5)	
Reason for PVC insertion		
Infusion	145 (67.4)	
Medication	53 (24.7)	
Blood product transfusion	17 (7.9)	
Administration method		
Gravitational	114 (53)	
Volume-controlled burette	53 (24.7)	
Infusion pump	48 (22.3)	
Fluids	58 (27)	
5% Dextrose	28 (13)	
10% Dextrose	21 (9.8)	
Normosaline solution	9 (4.2)	
Drugs (one or more)/device	145 (67.4)	
Number of drugs/device		
Antibiotics (one or more)/device	114 (53)	1 (1)
Aciclovir	7 (3.3)	
Paracetamol	31 (14.4)	
Corticosteroids	16 (7.4)	
Proton Pump Inhibitors	26 (12.1)	
Pamidronate	7 (3.2)	
Other	11 (5.1)	
Rhythm of device use/day (*N* = 165)		
≤3 times	92 (42.8)	
> 3 times	123 (57.2)	

*IQR* Inter-quartile range

### PVC complications

Complications were observed in 111 PIVCs (51.9%)in 46 children (46%), including infiltration in 80 cases (37.2%), accidental removal in 19 cases (8.8%), phlebitis in 11 cases (5.1%) and skin necrosis in one case. No local infection or device-related bloodstream infections were observed. Among the PIVCs with complications, 67 (60%) were replaced with a new device.

### Factors associated with PVC complications

Among the patient- and device-related variables considered in the univariate analysis, the following variables were significantly associated with the occurrence of local complications: a small catheter gauge (24-gauge) (*p* = 0.023), the use of the gravitational method, especially with a VCB (*p* = 0.036), a shorter indwelling time (*p* < 0.001), a longer duration of IV therapy (p < 0.001),medical diagnosis, especially respiratory and infectious diseases (*p* = 0.047), the type of IV drug used, especially antibiotics (*p* = 0.031), including cefotaxime(*p* = 0.024) and vancomycin (p = 0.031), and the use of proton pump inhibitors (PPIs) (*p* = 0.004). All of these variables were considered in the multivariate model and the use of PPIs (*p* = 0.011), a longer duration of IV therapy (*p* < 0.001) and a shorter indwelling time (p = 0.011) were found to be statistically significant.

None of the other variables, such as the ward, a patient’s age and gender, insertion site (in the hand, foot or head), the type of infusion, the administration of blood products, or the method of drug administration, contributed to the occurrence of local PVC complications. Table 3 shows the comparison of children and device characteristics in the two groups with and without complications.

**Table 3:** Comparison of children and device characteristics in the two groups with and without complications

Characteristics	Complication (*N* = 109)	No Complication (*N* = 106)	Univariate analysis		Multivariate Analysis
	Median ± IQR *N* (%)	Median ± IQR *N* (%)	p-value	HR [CI_95%_HR]	p-value
Male gender	59 (54.1)	57 (53.8)	0.958		
Age (years)	3 (5.7)	5 (6.7)	0.658		0.527
Duration of hospitalization (days)	13 (7)	8 (10)	0.093		0.383
Total duration of IV therapy/patient (days)	11(10)	7 (8.75)	<0.001		0.011
Dwell time (hours)	48 (48)	72 (51.2)	<0.001		<0.001
Rank of the PVC/patient	2 (2)	1 (1)	0.352		
Study site					
Pediatric Department A of CHBH	30 (27.5)	33 (31.1)	0.764		
Pediatric Department B of CHBH	14 (12.8)	10 (9.4)			
Pediatric Department C of CHBH	34 (31.2)	29 (27.4)			
Surgery Department A of CHBH	2 (1.8)	4 (3.8)			
Pediatric Department of LRH	29 (26.6)	30 (28.3)			
Diagnosis					
Infection	27 (24.8)	19 (17.9)	0.047		0.336
Respiratory	38 (34.9)	27 (25.5)			
Hematology	14 (12.8)	21 (19.8)			
Metabolic	9 (8.3)	21 (19.8)			
Other	21 (19.3)	18 (17)			
Site of PVC insertion					
Hand	86 (78.9)	85 (80.2)	0.0547		0.714
Foot	20 (18.3)	17 (16)			
Other	3 (2.8)	4 (3.8)			
Cannula gauge					
22G (blue)	50 (45.9)	65 (61.3)	0.023	1.87 [1.09–3.22]	0.164
24 G (yellow)	59 (54.1)	41 (38.7)			
Reason for PVC insertion					
Medication	76 (69.7)	69 (65.1)	0.656		0.769
Infusion	26 (33.9)	27 (25.5)			
Blood product transfusion	7 (6.4)	10 (9.4)			
Drug/infusion administration method					
Gravitational	52 (47.7)	62 (58.5)	0.036		0.216
Volume-controlled burette	35 (32.1)	18 (17)			
Infusion pump	22 (20.2)	26 (24.5)			
Administration rhythm ≥3 times/day	60 (55)	63 (59.4)	0.516		0.703
Number of medications/device	1 (1)	1 (1)	0.150		0.527
Antibiotics	68 (62.4)	46 (43.4)	0.005	2.16 [1.25–3.73]	0.615
Ampicillin	9 (8.3)	10 (9)	0.761		
Amoxicillin clavulanate	9 (8.3)	9 (8.3)	0.951		
Cefotaxim	29 (26.6)	15 (14.2)	0.024	2.19 [1.1–4.39]	0.545
Ceftriaxone	3 (2.8)	2 (1.9)	0.674		
Ceftazidime	9 (8.3)	7 (6.6)	0.644		
Fosfomycin	1 (0.9)	3 (2.8)	0.365		
Vancomycin	11 (10.1)	3 (2.8)	0.031	3.86 [1.04–14.29]	0.104
Amikacin	7 (6.4)	4 (3.8)	0.378		
Paracetamol	11 (10.1)	20 (18.9)	0.067		0.572
Aciclovir	5 (4.8)	2 (1.9)	0.244		
Proton Pump Inhibitors	20 (18.3)	6 (5.7)	0.004	3.74 [1.44–9.74]	0.011
Corticosteroids	10 (9.2)	6 (5.7)	0.326		
Pamidronate	2 (1.8)	5 (4.7)	0.275		
Infusate					
10% dextrose	10 (34.5)	11 (37.9)	0.445		
5% dextrose	16 (55.2)	12 (41.4)			
Normosaline	3 (10.3)	6 (20.7)			

*IQR* Inter-quartile range, *HR* Hazard ratio, CI95%HR: Confidence interval to 95% of the hazard ratio

When we considered each type of complication individually, some variables were associated with the occurrence of a specific type of complication in the multivariate analysis. Infiltration (or extravasation) was associated with a shorter indwelling time (*p* < 0.001), a longer duration of IV therapy (*p* = 0.007), the use of antibiotics, including ceftriaxone (*p* = 0.035) and vancomycin (*p* = 0.032), and PPI use (*p* = 0.005). Phlebitis was associated with PPI use (*p* = 0.031) and ceftriaxone (*p* = 0.017) and vancomycin (*p* = 0.011) use. Accidental PIVC removal was associated with a shorter indwelling time (p = 0.011).

### PIVC lifespan and associated factors

The cumulative lifespan of a PIVC was up to 15,011 h. The mean lifespan was 68.82 ± 35.71 h (range 1–168). The factors associated with PIVC lifespan were catheter gauge, the administration method, the rank of the IV device in each patient, the nature of the infusion and the study site. The mean lifespan was shorter for 24-gauge catheters (61.32 ± 33.25 h) compared to 22-gauge catheters (77.21 ± 36.26 h) (*p* < 0.001). When the gravitational method was used to administer infusions or drugs, the lifespan of the PIVC was longer (72.56 ± 35.85 h) when an electronic pump or syringe was used (60.03 ± 33.88 h) (p < 0.001). The lifespan of a PIVC decreased significantly with the rank of the device in the same patient (Pearson coefficient = −0.216, *p* = 0.001). The lifespan was shorter with the use of 10% dextrose (49.95 ± 28.70 h) compared to 5% dextrose (74 ± 39.39 h) and normal saline solutions (98.67 ± 47 h) (*p* = 0.005). Participants from the pediatric department of LRH had the shortest PIVC lifespan (57.14 ± 33.34 h) compared to the other departments (lifespan ranging from 65 ± 28.73 to 96 ± 33.94 h)(p = 0.005). When we considered the subgroup of PIVCs with complications, we found that the difference in lifespan between the different study sites was non-significant (*p* = 0.080). In the subgroup of devices without complications, the lifespan was significantly lower in the pediatric department of LRH(64.7 ± 32.56 h) than in the other wards (lifespan ranging from 86 ± 34 to 96 ± 33 h)(p = 0.001).

In the multivariate analysis, the use of 10% dextrose was the only factor associated with a reduced PIVC lifespan (*p* = 0.003). Table 4 shows the details of the comparison of the means and standard deviations PIVC lifespan between subgroups of patients and devices characteristics.

**Table 4:** comparison of PVC lifespan means and standard deviations between subgroups of patients and devices characteristics

	yes	no	p-value univariate	Spearman coefficient	p-value multivariate
	mean ± SD	mean ± SD			
Male gender	73.26 ± 36.88	65.84 ± 34.04	0.132		
Age (years)			0.612	−0.035	
Duration of hospitalization (days)			0.643	0.032	0.163
Total duration of IV therapy/patient (days)			0.551	0.041	
Rank of the PVC/patient			0.001	−0.216	0.509
Study site					
Pediatric Department A of CHBH	77.33 ± 35.66		0.005	0.160	0.019
Pediatric Department B of CHBH	65 ± 28.73				
Pediatric Department C of CHBH	73.52 ± 37.3				
Surgery Department A of CHBH	96 ± 33.94				
Pediatric Department of LRH	57.14 ± 33.34				
Diagnosis					
Infection	68.76 ± 33.47		0.109		0.582
Respiratory	72.31 ± 37.18				
Hematology	76.58 ± 34.27				
Metabolic	75.2 ± 36.31				
Other	56.71 ± 34.86				
Site of PVC insertion					
Hand	70.41 ± 36.04		0.590		0.068
Foot	66.91 ± 34.91				
Other	70.22 ± 35.54				
Cannula gauge24 G (yellow)	61.33 ± 33.25	77.21 ± 36.26	<0.001		0.610
Reason for PVC insertion					
Medication	71.16 ± 35.21		0.690		0.979
Infusion	66.21 ± 38.83				
Blood product transfusion	69.62 ± 30.62				
Use of infusion pump	72.56 ± 35.85	60.30 ± 33.88	0.009		0.233
Number of medications/device			0.614	0.041	0.330
Administration rhythm ≥3 times/day			0.220	0.004	0.444
Antibiotics	71.06 ± 37.45	68.42 ± 33.77	0.590		0.812
Ampicillin	67.89 ± 41.91	70.01 ± 35.17	0.806		
Amoxicillin clavulanate	65.33 ± 34.74	70.23 ± 35.85	0.579		
Cefotaxim	68.73 ± 33.38	70.1 ± 36.37	0.820		
Ceftriaxone	86 ± 33.49	69.43 ± 35.75	0.306		
Ceftazidime	79.25 ± 32.88	69.06 ± 35.89	0.273		
Fosfomycin	69.21 ± 35.43	102 ± 40.90	0.069		0.418
Vancomycin	66.86 ± 39.02	70.03 ± 35.56	0.749		
Amikacin	82.72 ± 20.22	69.12 ± 36.26	0.069		0.440
Paracetamol	86.71 ± 32.6	66.97 ± 35.5	0.004		0.164
Aciclovir	61.71 ± 23.42	70.31 ± 36.07	0.533		
Proton Pump Inhibitors	59.36 ± 34.29	71.26 ± 35.75	0.111		0.474
Corticosteroids	59.03 ± 28.06	70.69 ± 36.17	0.210		
Pamidronate	49.71 ± 20.85	70.50 ± 35.94	0.130		0.282
Infusate					
10% dextrose	49.95 ± 28.70		0.005		0.003
5% dextrose	74 ± 39.39				
Normal saline	98.67 ± 47				

*SD* Standard deviation, *IV* Intravenous, *PVC* Peripheral venous cannula, *CHBH* Children’s Hospital Béchir Hamza, *LRH* La Rabta Hospital

## Discussion

Local PVC complications were common in our pediatric departments, which were observed in approximately half of the devices and children. The complication rate was higher than those from other studies on comparable pediatric populations (not including neonatal and intensive care units) [11, 12]. These studies reported PVC complications in approximately25% of devices. The most frequent complications were infiltration and accidental removal, affecting 37.2% and 17.1% of devices, respectively. These rates were higher than the results of Malyon and al. who found rates of 14% and 5% for these complications [11] and de Lima Jacinto et al. who reported infiltration in 16% of children [12].

We noted one case of skin necrosis, which was a result of a late diagnosis of extravasation [28] partially due to a lack of monitoring. In the wards where the study was conducted, the nurse/patient ratio was variable, ranging from 1:4 to 1:10 and even as low as 1:13 to 1:20 during night shifts, affecting the quality of monitoring and increasing the risk of delayed diagnosis of complications and the occurrence of tissue damage [6].

The PIVC lifespan in our study was longer than that found in the literature (68.82 ± 35.71 (range 1–168)). Studies evaluating this variable in children are scarce. The mean lifespan in these studies ranges from 29.53 to 51 h, with significant variations (1 to 136 h) [6, 8, 17, 29, 30]. The PIVC lifespan was shorter with the use of 24-gauge catheters compared to 22-gauge catheters and electronic pumps or syringes compared to the gravitational method, a higher rank of the IV device in a patient, and the use of 10% dextrose solution compared to 5% dextrose and normal saline solutions. Only the use of 10% dextrose remained significant in the multivariate analysis. Gupta et al. studied the lifespan of PIVCs in a neonatal intensive care unit in India. He found no association between PIVC lifespan and the risk factors identified in our study. The use of cefotaxime was the only factor that reduced the lifespan of PIVCs with complications in this study [6]. No other studies have reported risk factors associated with a shorter PIVC lifespan, but all factors identified in our study were associated with PVC complications in the literature [12, 28].

Devices with complications had a shorter lifespan than those without complications. The literature on this subject is conflicting. De Lima Jacinto reported that complications were associated with a shorter indwelling time [12], while Malyon et al. found that indwelling time was longer in PIVCs exhibiting complications and investigated reasonable expectations regarding PIVC indwelling time in the acute pediatric population [11]. A recent review analyzed data from seven randomized controlled trials that compared routine removal of peripheral IV catheters with removal only when clinically indicated in hospitalized or community patients receiving continuous or intermittent infusions [31]. The authors found no evidence to support changing catheters every 72 to 96 h. Consequently, they suggested that healthcare organizations consider a policy in which catheters are changed only if clinically indicated.

We identified many risk factors associated with complications, but the ones that remained significant in the multivariate analysis were a longer duration of IV therapy, and the use of PPIs, ceftriaxone and vancomycin. Unlike other factors that have already been reported in the literature [6, 8, 12, 30, 32, 33], the association between the use of PPIs and the occurrence of PVC complications is a novel finding.

We did not find any studies reporting on or attempting to explain this finding. Our investigations after the study revealed that in four of the participating wards, administration recommendations for PPIs and other drugs were not respected. PPIs and corticosteroids were administered via direct bolus injection rather than slow infusion. Vancomycin was administered over 20–30 min, which is less than the recommended time (≥1 h). These practices were due to the lack of infusion pumps. The nurses reduced administration time to enable the use of the pumps in more patients. Therefore, one of the requirements to reduce complications in our pediatric departments is to have enough infusion pumps to administer medications in accordance with the recommendations and ensure the safe use of these devices [34].

The complication rate was significantly higher in the VCB subgroup compared to the standard gravitational drip and infusion pump subgroups. To our knowledge, this result has not been reported previously. This finding may be due to the lack of infusion pumps in our pediatric departments (1 pump/10–20 patients in each ward). Therefore, care providers tend to use VCBs rather than pumps to administer medications and infusions, especially in neonates and young infants. This device is indicated for short infusions (<24 h) with a 21-gauge cannula or larger. The main reported reason for complications related to this device is misuse [35]. In our study, VCBs were used with small gauge catheters and the mean duration of IV therapy with VCBs (14.91 ± 4.01 days) was longer than recommended and significantly longer compared with the gravitational method (7.62 ± 4.01 days) or infusion pumps (6.23 ± 4.54 days) (*p* < 0.001). In addition, the height of the infusion bag in relation to the patient must be 80 cm (Documentation technique ASEPT INMED. Régulateur de debit Dosi-Flow®). We found that these requirements were ignored among nurses in all wards that participated in our study.

Sixty percent of failed PIVCs were replaced, suggesting that the other 40% were unnecessary and could have been removed earlier. This result is consistent with the data from Malyon et al. who reported that only the half of failed PIVCs were replaced [11]. Limm et al. reported that half of PIVCs inserted in the emergency department was unused 72 h later [36].In the pediatric department of LRH, the lifespans of all PIVCs and those without complications were significantly shorter than those in other departments in CHBH. This difference was not observed in the devices exhibiting complications. Since routine PIVC replacement was not implemented in the participating wards, this result suggests that in the pediatric department of LRH, nurses tend to remove unused PIVCs earlier than in other wards. Therefore, the staff in these other wards needs to be educated regarding the removal of useless PIVCs to reduce the risk of PVC complications [11].

We also found that PVC complications were associated with a longer total duration of IV therapy. This is consistent with the data in the literature [12]. Rationalizing the use of IV therapy may contribute to reducing the PVC complication rate in our hospitals.

In our study, practices for PIVC insertion, dressing, safeguarding and maintenance were different from international standards [26]. Therefore, we considered that these practices, in addition to the other factors identified in our study, may have contributed to the higher rate of complications compared to the literature. In fact, it is known that PIVC dressings play a vital role in preventing catheter complications [37].

In our study, nurses used tape for PIVC dressings while Malyon et al. used bordered polyurethane dressings (BPU) [11]. Livesley and Richardson compared these two methods and found that peripheral venous catheter failure occurred less frequently in the tape group than in the bordered transparent dressing group [38]. However, replacing tape dressings with polyurethane dressings is not a priority for us in reducing the rate of PVC complications since polyurethane dressings are costly and unavailable in our hospitals.

Splinting was not routinely performed in our study. Laudenbach et al... found no differences in complication rates or types between two groups of children with and without splinting [39], while Tripathi et al. found that splints significantly prolonged PIVC survival, especially in younger patients [15]. Hugill et al. recommend the use of splints to reduce catheter movement, especially when a catheter is inserted in an area with high flexion and in neonates [25].We must consider the application of these recommendations to reduce the high rate of complications in our hospitals.

For skin preparation, alcohol 70% was used in our study, but chlorhexidine 2% in combination with alcohol 70% is recommended in international guidelines [34] and was used in Malyon et al’s study [11]. Studies comparing these two methods confirmed the superiority of chlorhexidine solution in reducing bacterial contamination or colonization on a device. No significant differences were noted in terms of displacement or infiltration between the two methods [40, 41]. The available data are not sufficient to determine whether the skin preparation procedure contributed to the relatively high rate of PVC failure in our study. Further investigations are required to determine whether to use chlorhexidine 2% in combination with alcohol 70% routinely, especially because this product is not available in all of our public hospitals. Recently, the use of chlorhexidine 2% in combination with alcohol 70% was introduced in CHBH. Therefore, a study should be conducted to evaluate the effect of this change in practice on the PVC-related complication rate in this hospital.

### Strengths and limitations

Our study is the first multicenter cohort study on PVC complications in a tertiary pediatric population in a developing country. Our results could be regarded as preliminary data for the implementation of practices to reduce the incidence of PVC complications in children in Tunisia and in other developing countries.

However, a few limitations exist in this study. The lack of funding limited the number of people dedicated to data collection. The two nurses who collected the data were volunteers, as this study was part of their end-of-study project, and they could only be present during business hours. This could be a source of bias as some patients, especially those who were admitted and released or transferred during night shifts, and rapidly removed devices could have been missed. To some extent, this could explain the longer mean lifespan of PIVCs in our study compared to other studies in children.

The variables studied were numerous, but because of limited feasibility due to limited resources for our study, we could not integrate all potential factors that could affect the occurrence of PVC complications, including details related to PIVC fixation and dressing, maintenance practices, and details regarding PIVC use (the sizes of the syringes used to administer each medication, the type of pump used, the infusion rate, and adherence to recommendations for each medication or device used) [15, 25, 39, 42–44]. These variables should be considered in future studies to evaluate actions that can be taken in our hospitals to reduce PVC-related complications.

## Conclusions

This study showed that the PVC-related complication rate is high in our pediatric departments. Many of the identified risk factors are preventable. Several practices can be implemented in the immediate future without additional cost, including rationalization of the use of this device, reducing the duration of IV therapy, removing unnecessary PIVCs, and better adherence to the recommendations for the use of each drug and each administration method. Improving PIVC protection using splints in smaller children and when devices are inserted in areas with high flexion should reduce infiltration and removal rates. Other actions, such as increasing the number of nurses per patient and supplying enough infusion pumps, require additional financial investments and the involvement of health authorities in our country. Further studies should be conducted to evaluate the effects of these actions on PVC complication rates in our hospitals.

## Abbreviations

CHBH: CHILDREN’S Hospital Béchir Hamza
CI_95%_: 95% confidence interval
HR: Hazard ratio
IQR: Interquartile range
IV: Intravenous
LRH: La Rabta Hospital
PIVC: Peripheral intravenous venous catheter
PPI: Proton pump inhibitors
PVC: Peripheral venous catheterization
VCB: Volume-controlled burette
