# Correction to: Full title: peripheral venous catheter complications in children: predisposing factors in a multicenter prospective cohort study

**DOI:** 10.1186/s12887-018-1281-x

**Published:** 2018-09-24

**Authors:** Rim Ben Abdelaziz, Habiba Hafsi, Hela Hajji, Hela Boudabous, Amel Ben Chehida, Ali Mrabet, Khadija Boussetta, Sihem Barsaoui, Azza Sammoud, Mourad Hamzaoui, Hatem Azzouz, Néji Tebib

**Affiliations:** 1 Department of Pediatrics, La Rabta Hospital, Tunis, Tunisia; 2 Faculté de Médecine de Tunis; LR12SPO2 les maladies héréditaires du métabolisme investigation et prise en charge, Université Tunis El Manar, Tunis, Tunisia; 3 Faculté de Médecine de Tunis; Military General Directorate of Health, department of Epidemiology and Public Health, Université Tunis El Manar, Tunis, Tunisia; 4 Université Tunis El Manar, Hôpital d’enfants Béchir Hamza de Tunis, Faculté de Médecine de Tunis; Department of Pediatrics B, Tunis, Tunisia; 5 Faculté de Médecine de Tunis; Department of Pediatrics A, Université Tunis El Manar, Hôpital d’enfants Béchir Hamza de Tunis, Tunis, Tunisia; 6 Faculté de Médecine de Tunis; Department of Pediatrics C, Université Tunis El Manar, Hôpital d’enfants Béchir Hamza de Tunis, Tunis, Tunisia; 7 Université Tunis El Manar, Hôpital d’enfants Béchir Hamza de Tunis, Faculté de Médecine de Tunis; Department of Pediatric Surgery A, Tunis, Tunisia; 8 Department of Pediatrics, La Rabta Hospital Jabbari, 1007 Tunis, Tunisia

## Correction

Following publication of the original article [1], one of the authors flagged that the title of the article was submitted (incorrectly) with “Full title:” at the beginning.

As such, please be advised that the correct title is: Peripheral venous catheter complications in children: predisposing factors in a multicenter prospective cohort study.

## References

[1] Ben Abdelaziz R, et al. Full title: peripheral venous catheter complications in children: predisposing factors in a multicenter prospective cohort study. BMC pediatrics. 2017;17:208. 10.1186/s12887-017-0965-y. 10.1186/s12887-017-0965-y PMC5735659 29258474

